# Innovative Mycotoxin Detoxifying Agents Decrease the Absorption Rate of Aflatoxin B1 and Counteract the Oxidative Stress in Broiler Chickens Exposed to Low Dietary Levels of the Mycotoxin

**DOI:** 10.3390/toxins17020082

**Published:** 2025-02-10

**Authors:** Matteo Cuccato, Neenu Amminikutty, Veronica Spalenza, Vanessa Conte, Stefano Bagatella, Donato Greco, Vito D’Ascanio, Francesco Gai, Achille Schiavone, Giuseppina Avantaggiato, Carlo Nebbia, Flavia Girolami

**Affiliations:** 1 Department of Veterinary Sciences, University of Torino, 10095 Grugliasco, Italy; matteo.cuccato@unito.it (M.C.); neenu.amminikutty@unito.it (N.A.); spalenza.veronica@gmail.com (V.S.); vanessa.conte@unito.it (V.C.); achille.schiavone@unito.it (A.S.); flavia.girolami@unito.it (F.G.); 2 Institute of Sciences of Food Production, Italian National Research Council, 70126 Bari, Italy; donato.greco@ispa.cnr.it (D.G.); vito.dascanio@ispa.cnr.it (V.D.); giuseppina.avantaggiato@ispa.cnr.it (G.A.); 3 Institute of Sciences of Food Production, National Research Council, 10095 Grugliasco, Italy; francesco.gai@ispa.cnr.it

**Keywords:** aflatoxin B1, mycotoxin detoxifying agents, oxidative stress, liver, chicken, drug-metabolizing enzymes

## Abstract

Aflatoxin B1 (AFB1) can impair the growth of chickens and reduce the quality of eggs and meat, resulting in significant economic losses. The inclusion of mycotoxin detoxifying agents (MyDA) with binding properties in the diet is an efficient tool to reduce their absorption rate in the gastrointestinal tract. Our aim was to investigate the ability of two innovative MyDA (SeOX, a feed additive featuring a tri-octahedral smectite mixed with lignocellulose, and CHS, a di-octahedral smectite functionalized with an organic non-toxic modifier) in both reducing the bio-accessibility and mitigating the adverse effects of AFB1 in broilers exposed for 10 days to concentrations approaching the European Union maximum limits in feed (0.02 mg/kg). The amount of AFB1 in the excreta of birds, collected over four consecutive days (starting on day 7), was significantly lower (*p* < 0.001) in the group exposed to AFB1 alone compared to the groups treated with either SeOX or CHS. The calculated bio-accessibility was decreased by nearly 30% with both MyDA. This positive effect was reflected by a significant reduction (*p* < 0.001) in the oxidative stress (measured as serum antioxidant capacity and hepatic lipid peroxidation) induced by AFB1. Although antioxidant enzyme activities and glutathione levels were unaffected by any treatment, AFB1 significantly induced (*p* < 0.001) the upregulation of CYP2A6 and the downregulation of Nrf2; the latter was reverted by each MyDA. Overall, these results demonstrate that the selected MyDA are effective in limiting the AFB1 absorption rate, thereby mitigating or even reverting the oxidative stress induced by AFB1 in broilers.

## 1. Introduction

Aflatoxins (AF) are secondary metabolites mainly produced by mycotoxigenic fungi of the genus Aspergillus [1]. AFB1 is one of the most extensively studied mycotoxins in humans and in a diverse range of animal species. It is widely acknowledged for its hepatotoxic and immunotoxic properties, and it has been classified as a Group I genotoxic carcinogen by the International Agency for Research on Cancer (IARC) [2]. In sub-tropical and tropical countries, AFB1 contamination in food and feed commodities has long been recognized as a significant public health issue [3]. However, the combination of rising temperatures and increased humidity caused by climate change in tempered zones has escalated the contamination into a global emergency [4]. In the poultry industry, feed contamination by AFB1 poses a substantial challenge [5]. Although chickens are relatively less sensitive to aflatoxicosis compared to the other avian species raised for food production (e.g., ducks and turkeys), the prolonged exposure of broilers to AFB1 can impair their growth performances, with reduced weight gain, feed intake, and feed conversion ratio [6,7]. Experimental studies have demonstrated that AFB1 can induce oxidative stress (OS) in various tissues and organs, thereby compromising the antioxidant defense system [8,9,10]. The main driver in the AFB1-mediated OS is aflatoxin-8,9-epoxide (AFBO), a bioactivated metabolite primarily generated by the phase I enzyme CYP2A6 ortholog in chickens, with CYP1A1 playing a secondary role. AFBO can be detoxified by reduced glutathione (GSH) via glutathione S-transferase (GST) and subsequently excreted as a mercapturic acid derivative. The phase I metabolism of AFB1 produces other less toxic oxidated water-soluble metabolites (i.e., AFM1, AFB2a, AFQ1, and AFP1) and a reduced form, aflatoxicol (AFL), which can be easily re-converted to the parent compound in liver [11].

Given the significant health risks posed by AF, regulatory bodies worldwide have established maximum permitted levels in food and feed. In the European Union (EU), the limits in both complementary and complete feed for farm animals range from 0.005 mg/kg for dairy ruminants to 0.02 mg/kg for the other food-producing species (EC Regulation 574/2011). However, similar regulatory restrictions have been implemented only in the USA and in a few African countries, and a homogenous global regulatory framework is still lacking [3]. Thus, minimizing the formation and accumulation of mycotoxins in crops remains crucial. AFB1 contamination can occur at any stage of the feed and food chain, from the field production to the final use of various plant products. Several control methods have proven effective in mitigating AFB1 accumulation in cereals from harvest to storage, ensuring compliance with EU regulatory limits [12]. Additionally, the inclusion of low-cost and easy-to-use technological feed additives, known as mycotoxin adsorbents or binding agents (e.g., clays, silicates, yeast cell walls, and probiotic bacteria), represents the prevalent approach to reduce the gut absorption of mycotoxins, thus counteracting their toxic effects [13].

According to a Scientific Opinion of the European Food Safety Authority (EFSA), mycotoxin-detoxifying agents (MyDA) are defined as compounds either capable of adsorbing mycotoxins, binding them to their surfaces, or transforming them into less toxic derivatives through biotransformation [13]. From a regulatory perspective, mycotoxin binders are classified as technological feed additives, and their use is authorized for food-producing animals (EC Regulations 1831/2003 and 786/2015). Currently, bentonite is the only authorized mycotoxin binder for reducing AFB1 absorption in pigs, poultry, and cattle (EC Regulation 1060/2013). According to the recently published literature, several commercial feed additives containing bentonite as the main compound have been shown to effectively sequester AFB1 and tackle its toxicity in chickens [14,15,16,17,18]. However, such inorganic binder rarely shows activity against other mycotoxins, such as T-2 toxins, ochratoxin-A, deoxynivalenol, and fumonisins [12]. In recent years, several authorized and experimental additives have been investigated for their ability to function as MyDA and mitigate the harmful effects of mycotoxins in poultry [19,20,21,22,23]. The most studied ones are typically based on mineral clays or other inorganic compounds that can trap mycotoxins from contaminated feed, thereby reducing their absorption in the gastrointestinal system and facilitating their fecal elimination [12]. Conversely, organic additives such as yeast cell wall, probiotic Lactobacilli strains, or *Saccharomyces cerevisiae* act by modifying the chemical structure of the mycotoxins and alleviating their adverse effects [24,25].

In previous published studies, broilers dietarily exposed to AFB1 at levels around the EU regulatory limits (0.02 mg/kg) showed significant hepatic, renal, and systemic OS [9,10]. Of note, dietary supplementation with turmeric powder reverted such effects and counteracted the negative modulation of selected target genes induced by AFB1. The concomitant reduction in hepatic AFB1 residues and the positive gene regulation of intestinal and hepatic transporters suggest the potential ability of turmeric powder to reduce intestinal AFB1 absorption or to increase its excretion. In general, the use of feed exceeding the EU limit for AFB1, even with the supplementation of an authorized MyDA, is not permitted in the EU Member States. To the best of our knowledge, most of the research on chickens has investigated the efficacy of commercial or innovative MyDA in counteracting the negative effects of feed contaminated by AFB1 at concentrations ranging from 0.1 to 10 mg/kg [20,22,26,27], thus always exceeding the EU regulatory limit for poultry feed. It remains to be established whether the use of MyDA in feed contaminated by AFB1 concentrations compliant with the EU limits may be effective in ameliorating the OS caused by such low levels of the mycotoxin in chickens [10].

Therefore, the aim of this study was to investigate the potential ability of two innovative MyDA, a smectite-based material (SeOX) and a bio-organoclay (CHS), to limit both the bio-accessibility and the adverse effects of AFB1 in broilers exposed to concentrations approaching the EU maximum limits in feed (0.02 mg/kg). SeOX was obtained by combining a tri-octahedral smectite with a lignocellulose-based material showing antioxidant properties, as described by Greco et al. [28]. The CHS was developed by functionalization of a di-octahedral smectite with an organic, non-toxic modifier. This product can be considered safe, as it was obtained using agents listed in the EU Register of Feed Additives (EC Regulation, No.1831/2003). Since the primary target organ of AFB1 toxicity is the liver [29], the hepatic effects of the exposure to the mycotoxin in the presence or absence of each MyDa were explored.

## 2. Results

### 2.1. Effects of AFB1 and MyDA on Zootechnical Parameters and Liver Histology

Throughout the trial, the birds of each experimental group maintained good health, and no mortality occurred. No statistically significant differences in the zootechnical parameters were registered as the result of the exposure to AFB1 alone or in combination with either SeOX or CHS; this was also true for chicks treated with each MyDA alone (Table 1). Additionally, no effects on the dry matter (DM) digestibility were observed in any of the treated groups, with an average value of 64.3%.

**Figure 1:**
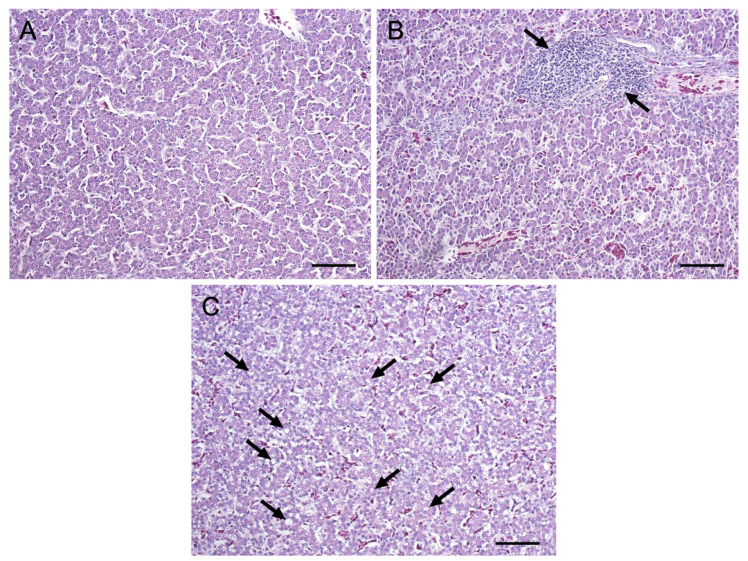
Representative histopathological examination of photomicrographs of broiler livers. (**A**) Absence of lesions from aflatoxin B1 (AFB1) group; (**B**) focal, moderate, and lymphoplasmacytic inflammation (arrows) from basal diet (BD) group; (**C**) mild vacuolar degeneration of hepatocytes (arrows) from AFB1 + SeOX group. Hematoxylin-eosin, 200X; scale bars: 50 µm.

No significant histological changes were observed in the hepatic tissue from animals of every experimental group. Mild to moderate multifocal lymphoplasmacytic hepatitis, accompanied by a multifocal lymphoid hyperplasia, and/or mild vacuolar degeneration of the hepatocytes were randomly distributed among birds (Figure 1). No significant differences were observed between broilers from the control and treated groups.

### 2.2. Effects of MyDA on the AF Content in Broiler Excreta and on the AF Absorption Rate

**Figure 2:**
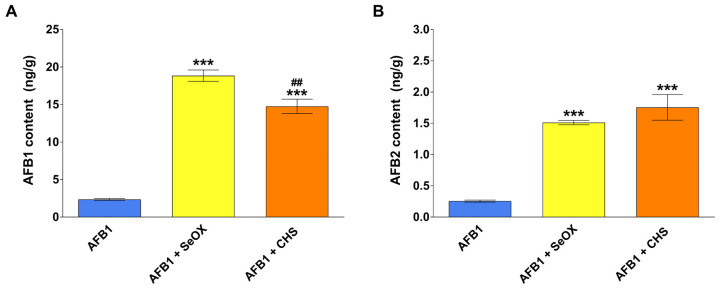
Concentrations of AFB1 (**A**) and AFB2 (**B**) in the excreta of broilers during the last 4 days of treatment. AFB1, aflatoxin B1 group; AFB1 + SeOX, aflatoxin B1 plus SeOX group; AFB1 + CHS, aflatoxin B1 plus CHS group. Each value represents the mean (±SEM) of four replicates analyzed in duplicate (n = 8) (*** *p* < 0.001 vs. AFB1; ## *p* < 0.01 vs. AFB1 + SeOX).

The content of AF (i.e., AFB1, AFB2, AFG1, and AFG2) and their metabolites (i.e., AFM1, AFM2, AFQ1, and AFL) was measured in the broiler excreta collected in the last 4 days of the treatment period by means of an optimized high-performance liquid chromatography with fluorescence detection (HPLC-FLD) method. The average amounts of AFB1 and AFB2 in the different experimental groups are shown in Figure 2. The excreta of both the SeOX and CHS-supplemented groups exhibited a 7- to 9-fold higher AFB1 concentration (*p* < 0.001) compared to the broilers that received the mycotoxin alone (2.3 ± 0.3 ng/g). Moreover, SeOX treatment proved to be slightly more efficient in reducing the absorption of AFB1 than CHS (*p* < 0.01). Despite the minimal amount of AFB2 present in the contaminated feed (1.4 ± 0.2 μg/kg), the toxin was detected in all samples. The excreta collected from the SeOX- or CHS-supplemented group exhibited 5- to 6-fold higher AFB2 values compared with chickens fed the contaminated diet alone (0.3 ± 0.04 ng AFB2/g), with no statistical differences between each MyDA. With regard to the remaining molecules, AFG1, AFG2, AFM2, and AFQ1 were not detected in any of the samples, whereas AFM1 and AFL were present at relatively low levels (below the limit of quantification, LOQ).

**Figure 3:**
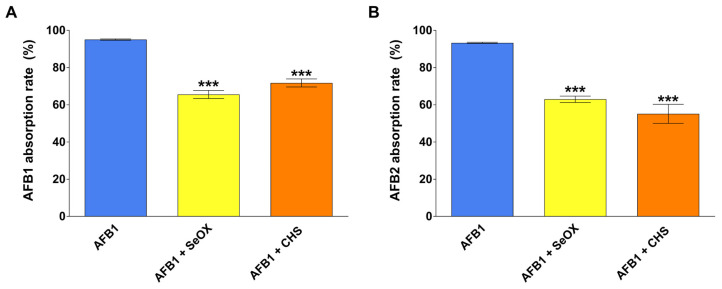
Absorption rate of AFB1 (**A**) and AFB2 (**B**) calculated on the dry matter (DM) and AF concentration of both feed intake and excreta (see details in the M&M section). AFB1, aflatoxin B1 group; AFB1 + SeOX, aflatoxin B1 plus SeOX group; AFB1 + CHS, aflatoxin B1 plus CHS group. Data are expressed as mean ± SEM, n = 8 (*** *p* < 0.001 vs. AFB1).

The mycotoxin absorption rate is depicted in Figure 3. In the AFB1 group, there was an extensive absorption rate for both AFB1 and AFB2 (close to 95%), which was significantly reduced (by about 30%, *p*< 0.001) in both AFB1 + SeOX and AFB1 + CHS groups. No statistically significant differences in the ability to reduce the AFB1 absorption rate were detected between SeOX and CHS.

### 2.3. Effects of AFB1 and MyDA on Serum Antioxidant Capacity (SAC) and Hepatic Lipid Peroxidation

**Figure 4:**
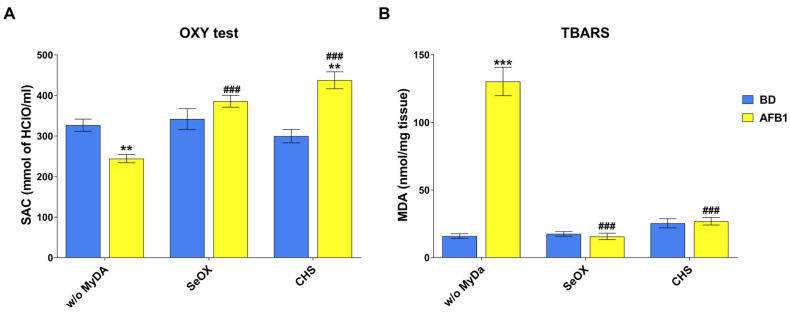
Effects of AFB1 and MyDA on SAC (**A**) and hepatic lipid peroxidation (**B**) in broilers at the end of the treatment (10 days). BD, basal diet; AFB1, BD + aflatoxin B1; w/o MyDA, diet not supplemented with MyDa; SeOX, SeOX-supplemented diet; CHS, CHS-supplemented diet. Data are expressed as mean ± SEM, n = 8 (** *p* < 0.01 vs. BD w/o MyDA; *** *p* < 0.001 vs. BD w/o MyDA; ### *p* < 0.001 vs. BD + AFB1 w/o MyDA).

The measurement of SAC by the OXY test was performed to assess the systemic antioxidant response of broilers to the different treatments. As shown in Figure 4A, a 10-day exposure to AFB1-contaminated feed reduced the SAC by approximately 30% in comparison with the control group (*p* < 0.01). Neither the binder SeOX nor CHS alone affected the SAC of broilers. Conversely, when AFB1 was administered together with either of the two MyDA, the co-treatment induced a statistically significant increase in SAC (*p* < 0.001), reverting the pro-oxidant effect of AFB1 and restoring the antioxidant defense of the birds to control or even higher values (i.e., in the presence of CHS).

MDA levels were assessed in liver samples by the Thiobarbituric Reactive Substances (TBARS) test to investigate the lipid peroxidation. An increase in MDA content by approximately 6-fold was observed in AFB1-treated broilers in comparison with the control group (Figure 4B, *p* < 0.001). Neither of the two MyDA alone affected the hepatic MDA levels. In contrast, the administration of AFB1 with either SeOX or CHS resulted in a statistically significant reduction in MDA content to control values, reverting the negative effects of the mycotoxin (*p* < 0.001).

### 2.4. Effects of AFB1 and MyDA on the Expression of Selected Hepatic Genes

**Figure 5:**
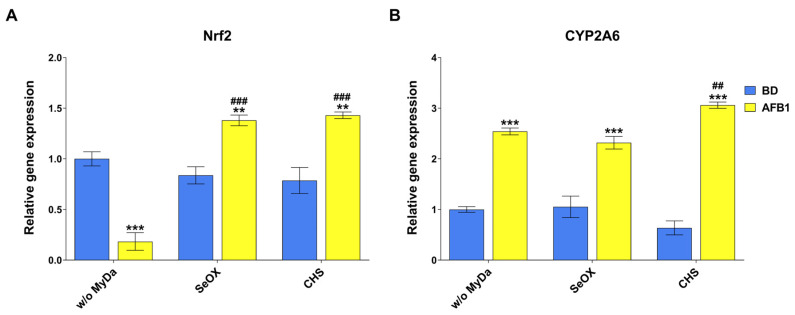
Effects of AFB1 and MyDA on the mRNA expression of Nrf2 (**A**) and CYP2A6 (**B**) in the liver of broilers at the end of the treatment period (10 days), measured using qRT-PCR. BD, basal diet; AFB1, BD + aflatoxin B1; w/o MyDA, diet not supplemented with MyDa; SeOX, SeOX-supplemented diet; CHS, CHS-supplemented diet. Data are expressed as mean ± SEM, n = 8 (** *p* < 0.01 vs. BD w/o MyDA; *** *p* < 0.001 vs. BD w/o MyDA; ## *p* < 0.01 vs. BD + AFB1 w/o MyDA; ### *p* < 0.001 vs. BD + AFB1 w/o MyDA).

The analysis of mRNA expression in liver was conducted on a panel of genes encoding for (i) enzymes involved in AFB1 metabolism (i.e., CYP1A1, CYP1A2, CYP2A6, CYP2H1, CYP3A4, EPHX1, EPHX2, all isoforms of GSTA, and GSTM2), (ii) the antioxidant defense pathway (i.e., Nrf2, CAT, GPX1, SOD1, and SOD 2), and (iii) drug transporters (DT) responsible for AFB1 efflux (i.e., ABCB1, ABCC2, and ABCG2). While all genes were expressed across all samples, only CYP2A6 and Nrf2 were significantly modulated in response to the treatments (Figure 5). Specifically, Nrf2 was downregulated by approximately 5-fold in the AFB1 group, but the concomitant administration of each MyDA successfully restored Nrf2 expression to control levels (Figure 5A). Conversely, CYP2A6 was upregulated by approximately 2.5-fold by AFB1 in comparison with the control group; however, the co-treatment with either SeOX or CHS was not able to mitigate such effect (Figure 5B).

### 2.5. Effects of AFB1 and MyDA on Hepatic GSH Content and Enzymatic Activities

Determination of GSH content and other enzymatic activities (total GST, µ-class GST, α-class GST, and DT-diaphorase) was conducted in liver to investigate the role of AFB1 and the selected MyDA in the modulation of the main detoxification pathways involved in AFB1 metabolism. None of the investigated enzymatic activities and the GSH content, shown in Table 2, were modulated either by AFB1 or by the other treatments.

## 3. Discussion

In the poultry industry, MyDA are widely used to mitigate the negative effects of mycotoxin feed contamination. These agents often trap mycotoxins in the gastrointestinal tract, thereby reducing their bio-accessibility and preventing or limiting their toxic effects. AFB1 has been demonstrated to induce OS and other adverse effects in the liver and kidney of broilers exposed to levels around the EU regulatory limit [9,10]. Most published studies have investigated the efficacy of MyDA using much higher dietary concentrations of AFB1, typically ranging from 0.1 to 10 mg/kg [26]. According to the European legislative framework, the abovementioned scenario is unrealistic since the use of feed with such AFB1 contamination is not allowed in routine poultry farming. Therefore, this study aimed to investigate the efficacy of two innovative MyDA in limiting both the bio-accessibility and the adverse effects of AFB1 in broilers exposed to concentrations approaching the EU maximum limits for chicken feed.

Smectite-based MyDA have long been extensively employed to promote AFB1 sequestration in animal feed [30]. The efficacy of SeOX as a multi-mycotoxin adsorbing agent (i.e., AFB1, fumonisin B1, ochratoxin A, zearalenone, and T-2 toxin) has been previously demonstrated through a series of in vitro studies including adsorption isotherms. The additive proved effective to almost completely adsorb AFB1 at both gastric and intestinal pH values [28]. Likewise, the CHS MyDA at low concentrations (0.25–0.5% w/v) successfully sequestered in vitro more than 95% of AFB1 and other mycotoxins in a large range of pH values (3–9) (unpublished data).

As previously reported [9,31,32], the schedule of AFB1 administration used in this trial (0.02 mg/kg diet for 10 days) neither caused mortality nor affected zootechnical performances and diet digestibility. In addition, it did not induce any microscopical changes in hepatic tissue [10] despite the occurrence of OS as evidenced by the increase in liver TBARS. To the best of our knowledge, the treatment protocol that can induce initial histological changes in chicken liver is 0.05 mg/kg diet of AFB1 for 28 days [7]. Nevertheless, it is conceivable that the OS generated by the low doses used in this study may result in overt histological changes upon a longer exposure [33]. It is also noteworthy that neither SeOX nor CHS alone (0.5% dietary supplementation) affected the growth performances or health of broilers, in line with a substantial body of data indicating that smectite-based MyDA are free of negative effects in poultry when applied at dietary concentrations like those used in this study [30].

Most of the available literature on the efficacy of binders focuses on their ability to mitigate the adverse effects (endpoints) caused by the mycotoxins. For AFB1, the most common endpoint is the impairment of growth performances [30], which in our study was not affected by the treatment schedule. Another method to demonstrate the in vivo efficacy of a MyDA acting as a binder is the measurement of the mycotoxin concentration in the excreta in the absence or presence of the additive [34]. In a trial where chickens were fed a diet containing AFB1 at concentrations similar to those used in our study (0.018 mg/kg), the AFB1 and AFB2 content in the excreta after 14 days was superimposable to our study, amounting to 2.3 ± 1.6 ng/g and 0.2 ± 0.1 ng/g, respectively [35]. The significant increase in the content of both AFB1 (7- to 9-fold) and AFB2 (5- to 6-fold) that we observed in the excreta from SeOX- or CHS-supplemented chickens aligns with the sequestration of the AF in the gastrointestinal tract promoted by both additives. When administered at low levels to chickens, AFB1 is extensively bio-transformed into several more polar metabolites (e.g., AFM1, AFBQ1, AFBP1, and AFL), which, after undergoing phase II metabolism, may be excreted via the biliary and urinary routes [36]. After absorption, AF cannot be excreted as such in avian and mammalian species unless they undergo both oxidative and conjugative metabolism [37]. Therefore, it may be assumed that the amount of AFB1/AFB2 recovered in the excreta likely represents the unabsorbed toxins. Based on these data, the supplementation of SeOX or CHS at inclusion levels of 0.5% led to a decrease in AFB1/AFB2 bio-accessibility up to 30%, promoting their elimination via the excreta. Our findings are difficult to compare with the existing literature due to limited available data. For instance, a mixture of bentonite and sepiolite fed to chicks at an inclusion level of 0.1% resulted in a two-fold increase in the content of AFB1 in the excreta with respect to chicks exposed to the mycotoxin alone (0.01 mg/kg diet) [34].

Only sparse information is available on the effects of MyDA on AFB1-mediated OS. As regards SAC, the increase to control values elicited by either SeOX or CHS in the AFB1 co-treated groups is similar to the findings of Khanian and colleagues [38], who described the same effect using a *Lactobacillus plantarum 299v* probiotic strain in broilers fed a 0.2 or 2 mg AFB1/kg contaminated diet for 42 days. The use of natural antioxidants to restore SAC was also successfully exploited by turmeric powder in broilers exposed to 0.02 mg AFB1/kg feed for 10 days [9] or by vitamin E [26]; in the latter case, the restoration of blood oxidative resistance in laying hens treated for 3 weeks with increasing doses of AFB1 (from 0.1 to 10 mg AFB1/kg diet) occurred only at AFB1 concentrations lower than 3 mg/kg.

Further protective effects of SeOX and CHS against AFB1-mediated OS were also observed for the TBARS assay, with the hepatic MDA levels being comparable to control values. Liver MDA values similar to controls were also documented in broilers receiving a diet containing 0.25 mg/kg of AFB1 for 42 days, supplemented with a mixture of aluminosilicates and glucomannans [39]. The increase in MDA content detected in the blood and kidney but not in the liver of broilers treated with diets containing 0.1 mg/kg AFB1 for 21 days was fully counteracted by the addition of zeolite [20].

For a more comprehensive understanding of the interactions between AFB1 and the selected MyDA, a gene expression analysis was conducted on genes encoding for enzymes involved in AFB1 bioactivation/detoxification but also related to OS modulation and drug transport. In the current study, none of the investigated genes (Table S1) were modulated by any of the different treatments except for CYP2A6 and Nrf2. As regards CYPs, most of the published literature on avian species focused on the effects of AFB1 and MyDA on liver CYP1A1, CYP1A2, CYP2H1, and CYP3A4. Studies in chickens and ducks generally suggest an AFB1-mediated downregulation of these CYPs, which is partially restored upon MyDA supplementation [15,39,40]. Although these CYPs contribute to some extent to AFB1 oxidative bio-transformations, CYP2A6 is consistently identified as the key enzyme involved in AFBO generation in chickens, which leads to pro-oxidant and cytotoxic effects [11]. Despite the low AFB1 exposure level and the short treatment duration, we observed an almost 2.5-fold upregulation of CYP2A6, in line with other studies performed with the administration of higher AFB1 dosages. For instance, the inclusion of either 0.5 mg/kg or 5 mg/kg of AFB1 in broiler feed for 21 or 28 days increased CYP2A6 expression by approximately 1.5- and 2.5-fold, respectively [41,42]. The AFB1-mediated increased expression of liver CYP2A6 is expected to enhance the generation of AFBO, triggering an overall worsening of the adverse effects in exposed birds. Contrary to what was observed for other parameters, the supplementation of either SeOX or CHS failed to counteract the increase in hepatic CYP2A6 expression caused by AFB1. These results are difficult to interpret and suggest that liver CYP2A6 induction is one of the most sensitive biomarkers of AFB1 exposure in chickens. It is worth noting that turmeric powder, curcumin, and curcuminoids were effective in counteracting the AFB1-related increase in hepatic CYP2A6 in several in vivo experiments performed in chickens [42,43,44].

Nrf2, a key transcription factor regulating the cell response to OS, underwent downregulation by AFB1, which is considered a feature of the pro-oxidant mechanisms displayed by the mycotoxin in different species, tissues, and cell systems [45]. However, in our study, the inclusion of each mycotoxin binder in the AFB1-contaminated diet resulted in an increase in Nrf2 expression, with levels even slightly higher than those observed in control animals. For comparison, the hepatic Nrf2 downregulation detected in broilers treated with 0.1 mg AFB1/kg diet for 14 days was not reversed by the addition of zeolite [20]. It is noteworthy that when SeOX or CHS were added alone to the control diet, they failed to modulate Nrf2 expression, pointing to a lack of systemic effects of the tested MyDA. In our study, the AFB1-mediated Nrf2 downregulation was not associated with a modulation of OS genes (i.e., SOD1, SOD2, CAT, or GPX1), as previously documented in chickens treated with higher doses of AFB1 (0.33 mg/kg for 21 days [46], 0.75 mg/kg for 30 days [47], or 1 mg/kg for 21 days [48]) According to the published literature, the consequences of Nrf2 downregulation in chickens are not yet fully understood. Given the complexity of the Nrf2 pathway, alternative genes may be regulated instead of the major ones commonly investigated, such as SOD, CAT, and GPX [49].

GST enzymes, especially those belonging to the α- and µ-class families, are responsible for AFB1 detoxification through the conjugation of AFBO with GSH [11]. An additional enzyme involved in the regulation of AFB1-mediated OS is DT-diaphorase, which has been shown to be downregulated by AFB1 in the liver of various animal species, including chickens, ducks, and rabbits [50,51,52]. Limited data exist on the hepatic modulation of GSTs and DT-diaphorase activities in response to low AFB1 exposure [31], and there is scant information on how MyDA affect these parameters. In our study, liver GSH content, GST activities related to α- and µ-class families, and DT-diaphorase activities were not modulated in broilers exposed to 0.02 mg AFB1/kg diet for 10 days. None of the above parameters were affected by either SeOX and CHS alone or in combination with AFB1. Our findings are consistent with previous investigations, which also found that AFB1 (0.1 mg/kg diet for 21 days) did not change hepatic GSH content and GSH-peroxidase activity in broilers even in the presence of zeolite [20].

In our study, the observed increase in liver TBARS did not coincide with a reduction in GSH content. Although these results are difficult to interpret, controversial effects of AFB1 on liver GSH and TBARS have been reported in the limited studies conducted on chickens. The increase in liver TBARS content was not matched by a decrease in GSH content, which remained unaltered, in broilers exposed to 17 µg AFB1/kg diet for 21 days [20]. In another study, the treatment of broilers with higher AFB1 dietary concentrations (100 µg/kg diet for 14 days) resulted in a limited decrease in hepatic GSH content (−30%) along with an increase in TBARS [44]. In contrast, neither liver TBARS nor liver GSH were altered by the repeated exposure of broilers even to higher dosages (150 µg/kg diet for 14 days) [53].

A number of potential limitations of this study should be mentioned. In some instances (CYPs, Nrf2, and some antioxidant enzymes), only the effects on gene expression were investigated. However, it should be noted that the main aim of this study was to assess the ability of two different MyDA to reduce the bio-accessibility and the enteric uptake of AF (as it was the case), thereby positively affecting the expression of target genes known as biomarkers of AFB1 toxicity. Secondly, the trial was conducted using a single dietary concentration of both AF and MyDA. It might be interesting, for instance, to test the efficacy of lower SeOX or CHS concentrations in the presence of the same AF dietary levels used in this study; alternatively, the binding ability of the additives might be assessed with AF concentrations in feed higher than those permitted in the EU, as is the case for most maize samples from many African countries [3].

## 4. Conclusions

The dietary exposure of chickens to the EU maximum allowed AF concentration in poultry feed (0.02 mg/kg) for 10 days did not affect either the zootechnical performances or the histological morphology of liver. However, the treatment elicited systemic and hepatic OS as well as pro-oxidant changes in liver (i.e., increase in CYP2A6 and decrease in Nrf2 gene expression). The dietary inclusion (0.5%) of either SeOX or CHS, two novel smectite-based MyDA, was able to mitigate or even reverse most of the AFB1-mediated adverse effects mentioned above, conceivably by decreasing the bio-accessibility of the mycotoxin up to 30%. Interestingly, when administered alone, no negative effects were detected on zootechnical or any of the other tested endpoints. Further research is warranted to investigate the efficacy of SeOX or CHS supplementation in the presence of higher dietary AF concentrations and to assess possible interferences with the supply of nutrients, micronutrients, or feed additives [54].

## 5. Materials and Methods

### 5.1. In Vivo Trial and Samples Collection

The in vivo trial was authorized by the Institutional Animal Care and Ethic Committee of the University of Turin, Italy (Approval number = 319508/2017-PR), and performed according to Directive 2007/43/EC in the farm facilities of the Department of Veterinary Sciences in Turin, as previously described [9,10]. In brief, a total of 48 male, 18-day-old broiler chickens (ROSS 308, average weight 752 ± 26 g) were randomly allocated to six experimental groups (n = 8 animals/group; 2 birds per cage): basal diet (BD) group; AFB1 group (BD + 0.02 mg/kg feed AFB1); SeOX group (BD + 0.5% SeOX); CHS group (BD + 0.5% CHS); AFB1 + SeOX group (BD + 0.02 mg/kg feed AFB1 + 0.5% SeOX); and AFB1 + CHS group (BD + 0.02 mg/kg feed AFB1 + 0.5% CHS). Details on the preparation of the AFB1-contaminated feed can be found in Damiano et al. (2022) [9]. Briefly, a BD containing 5.0 ± 1.3 μg/kg of AFB1 and 0.9 ± 0.1 μg/kg of AFB2 (mean ± SD, n = 3) was artificially contaminated to reach 18.8 ± 3.7 μg/kg of AFB1 and 1.4 ± 0.2 μg/kg of AFB2 (mean ± SD, n = 5). The different diets were administered for 10 consecutive days, from 23 to 32 days of age. Water and feed were provided ad libitum, and overall growth performances were recorded. During the last 4 days of the trial, 24 h excreta samples were collected from each cage, according to Schiavone et al. [55]. Then, the excreta were cleaned from feathers and any other foreign matter and weighed. A single sample was prepared for each replicate (n = 2 animals) by pooling representative aliquots (20%) of the daily excreted weight over the last 4 days. Subsequently, a representative sample of the entire pool (cage) was frozen and freeze-dried before determining the toxin content. At the end of the treatment (day 10), blood from each bird was sampled from the brachial vein. Then, the animals were euthanatized by sodium pentobarbital overdosage, and liver samples were collected from each broiler, cut into aliquots and properly stored for the subsequent analyses. For the histological investigation, specimens were fixed in a 10% buffered formalin solution; for the determination of GSH content and lipid peroxidation and for the enzymatic activities assays, they were immediately frozen in liquid nitrogen and then stored at −80 °C. For the gene expression study, the samples were kept in RNAlater^®^ stabilization solution (Sigma-Aldrich, Milan, Italy) for 24 h and then stored at −80 °C until analysis.

### 5.2. DM Digestibility and Mycotoxin Absorption Rate Calculation

The DM digestibility was calculated according to the standard protocol reported by Schiavone et al. [55] as given below:

DM digestibility (%) = (DM feed intake − DM excreta)/DM feed intake × 100

Then, the mycotoxin absorption rate was calculated as follows:

Mycotoxin absorption rate (%) = [(DM feed intake × Mycotoxin_feed_) − (DM excreta × Mycotoxin_excreta_)]/(DM feed intake × Mycotoxin_feed_) × 100

where Mycotoxin_feed_ and Mycotoxin_exreta_ represent the mycotoxin (AFB1 or AFB2) concentration (ng/g DM) in feed and excreta, respectively.

### 5.3. Histopathological Examination

The formalin-fixed liver samples were embedded in paraffin according to the standard protocol, cut into sections of 5 μm thickness, and stained with traditional hematoxylin–eosin dye. Two sections from each chick were blinded-analyzed by two different pathologists under a light microscope.

### 5.4. Chemical Analysis of AF and Their Metabolites in Broiler Excreta

Unless mentioned otherwise, all reagents were of analytical grade. Acetonitrile and methanol were purchased from Carlo Erba Reagents srl (Milan, Italy). The water was of Milli-Q^®^ quality (Millipore, Bedford, MA, USA). The β-glucuronidase/aryl sulfatase enzyme solution (30/60 U/mL) was acquired from Merck KGaA (Darmstadt, Germany). AF standards in powder form were provided by Fermentek Ltd. (Jerusalem, Israel), with the exception of AFQ1 that was supplied by Cfm Oskar Tropitzsch GmbH (Marketredwitz, Germany). Stock solutions of each standard (1 µg/mL in acetonitrile) were kept in the dark at 4 °C. AflaTest™ WB immunoaffinity columns (IMA) were supplied by VICAM (Waters Corporation, Milford, MA, USA).

The method to analyze AF in broiler excreta was optimized and validated in-house. For the optimization study, “blank” chicken excreta, collected from animals fed AF-free diets, were artificially spiked with a known amount of AF and analyzed using various extraction procedures. These procedures differed in specific operational conditions, including (i) type and volume of solvents, (ii) extraction mode (blending and/or shaking), and (iii) extraction time. Furthermore, the clean-up process using immunoaffinity columns was enhanced by testing different types and volumes of washing solvents. The combination of operational parameters that provided the highest extraction yield was selected as the definitive method for determining AF in chicken excreta. This optimized method was validated by assessing the following parameters: selectivity, intermediate precision, recovery, linearity, limit of detection (LOD), and limit of quantification (LOQ). Under optimized experimental conditions, 5 g of lyophilized excreta were mixed with 1 g of NaCl and 50 mL of an extraction solvent consisting of acetonitrile and water in a 70:30 (*v/v*) ratio. The suspension was homogenized for 2 min using a blender and then shaken (170 rpm) at room temperature for 1 h. After AF extraction by shaking, the suspension was filtered through paper (Whatman^®^ Grade 4, Maidstone, UK), diluted 10 times with ammonium acetate buffer (0.1 M, pH 4.8), and incubated overnight with 25 μL of the β-glucuronidase/arylsulfatase (30/60 U/mL) solution. The extract was filtered again using glass microfiber filters (Whatman^®^ Grade GF/A) and cleaned up (20 mL) using AflaTest^®^ WB columns. Following the extract elution (approximatively 1 drop per second), a washing step was performed by 30 mL of PBS to remove impurities. AF were eluted from the columns with 2 mL of methanol and collected into a 4 mL amber silanized vial. The cleaned-up sample was concentrated by evaporation under an air stream at 40° C, and the residual AF was reconstituted with 1 mL of a mixture containing methanol/water (50:50, *v*/*v*). A volume of 100 μL was injected into a HPLC-FLD system (Agilent 1100 series coupled with the UVE™ system for post-column photochemical derivatization) for the detection of AF according to the method described by Amminikutty et al. [10]. The AF content is expressed as ng/g excreta. The validation of the method was performed according to Malachová et al. [56]. The linearity of the method was evaluated by analyzing five standard calibrants (prepared in methanol/water, 50/50, *v/v*) containing the eight AF (i.e., AFB1, AFB2, AFG1, AFG2, AFM1, AFM2, AFQ1, and AFL) at concentrations ranging from 0.1 to 25 ng/mL, which correspond to the contamination levels of poultry excreta from 0.5 to 125 ng/g. Matrix-matched calibrants were prepared by spiking the methanolic eluate of uncontaminated excreta samples with different amount of AF stock solutions according to the desired concentration. Standard and matrix-matched calibrants (both analyzed in triplicate) were used to prepare the standard and the matrix-matched calibration curves, respectively. The two calibration curves were used to calculate the signal suppression/enhancement (SSE%) as described by Amminikutty et al. [10]. As shown in Table S2 the matrix-matched calibration curves showed good determination coefficients (R^2^ > 0.998), although the goodness of fit (linearity) was assessed by the lack-of-fit test (*p* > 0.05). In addition, SEE% values between 92% and 102% confirmed the efficacy of the optimized protocol in separating interfering matrix compounds from AF. The LOD and the LOQ, expressed as ng AF/g excreta, were evaluated as a signal-to-noise ratio (S/N) equal to 3 and 10, respectively (Table S3). The low LOQ values indicated that the optimized method, in combination with the use of sensitive HPLC equipment, was highly sensitive in detecting AF in a complex matrix such as broiler excreta. Recovery values were determined by spiking uncontaminated fecal samples at 5 and 25 ng/g. The recovery and precision values (RSD%) were assessed by analyzing three replicates at each spiking level. Recovery values ranging from 101% to 112% were considered acceptable (Table S4). The calculated RSD% values were in the range of acceptability at ≤6% (EC Regulation 519/2014).

### 5.5. SAC Measurement

The SAC was assessed by the OXY-Adsorbent test from Diacron (Grosseto, Italy), according to the modified method previously described [57]. Briefly, 2 μL of serum (1:100 (*v*/*v*) dilution in MilliQ water) was incubated with 200 μL of HClO solution for 10 min at 37 °C in a 96-well plate. Then, 2 μL of N, N-diethyl-p-phenylenediamine was added, and the absorbance was read at 505 nm by a spectrophotometer. The intensity of the chromogenic complex is negatively correlated to the SAC. Data are expressed as mmol of HClO per mL.

### 5.6. TBARS Assay

The hepatic lipid peroxidation was determined by a modified version of the TBARS test [58]. Briefly, liver homogenates were obtained by disrupting the samples through the Tissue Lyzer LT (Qiagen, Hilden, Germany) for 5 min at 50 Hz in a buffer comprising 200 μL of NaCl (0.9% *w/v*), 200 μL of TCA (10% *w*/*v*), and 4 μL of butylated hydroxytoluene (BHT) (2% *w/v*). After centrifugation at 13,000× *g* for 15 min at 4 °C, supernatants, blanks, and/or MDA standard solutions were mixed with 400 μL of TBA solution (15% TCA (*w*/*v*) in glacial acetic acid, 0.38% TBA (*w*/*v*), and 0.25 N hydrochloric acid) and vortexed for 1 min. The development of MDA-TBA adducts was obtained by immersing the samples in a 95 °C water bath for 60 min and then cooling them in a cold-water bath for 10 min. After centrifugation at 13,000× g for 15 min, the absorbance of supernatants was read at 532 nm. All samples, blanks, and the MDA standard curve were analyzed in triplicate. Data are expressed as nmol of MDA per mg of tissue.

### 5.7. RNA Extraction and qRT-PCR

The gene expression analysis was performed as reported by Amminikutty et al. [10]. Briefly, total hepatic RNA was extracted using the Maxwell RSC simplyRNA Kit (Promega, Madison, WI, USA) following to the manufacturer’s protocol, and RNA quantitation was obtained through the NanoDrop ND-2000 UV-Vis spectrophotometer (Thermo Fisher Scientific, Waltham, MA, USA). To check RNA purity, the 260 and 280 nm ratio was measured, and all analyzed samples had values > 1.9. An automated electrophoresis station (Experion Instrument, Bio-Rad, Hercules, CA, USA) was used to verify the RNA Integrity Number (RIN), which was >7 for all samples. Next, reverse transcription of 1 μg of total RNA from each sample in a final volume of 20 μL was performed through the iScriptTM cDNA Synthesis Kit (Bio-Rad, Hercules, CA, USA) following the manufacturer’s instructions in the GeneAmp PCR System 9700 (Perkin Elmer, Waltham, MA, USA). The primers were picked on the *Gallus gallus* GenBank database and verified on the Ensembl mRNA sequences using Primer3 software (version 3.0, Applied Biosystems, Foster City, CA, USA). To avoid the amplification of contaminant genomic DNA, primers were selected at the exon/exon boundaries and tested for hairpin and dimer formation with the NetPrimer tool (available at http://www.premierbiosoft.com/netprimer/index.html, access date: 22 July 2024). Finally, the primers were validated through the BLAST tool (Basic local alignment search tool; https://blast.ncbi.nlm.nih.gov/Blast.cgi, access date: 17 June 2024). All primer information is reported in Table S1. PGK2 and RPS7 were used as housekeeping genes (HKG) according to Amminikutty et al. [10]. qRT-PCR was performed through the Bio-Rad CFX Opus 96 (Bio-Rad) at the following conditions: 30 s at 95 °C, 40 cycles of 5 s at 95 °C, and 30 s at 60 °C. Intra-assay variability was reduced by running each reaction in triplicate; water instead of cDNA was used as a blank control. An inter-run calibrator comprising a pool of cDNAs from control samples was included to check the inter-assay variation. Gene expression results were analyzed using the CFX Maestro software (Bio-Rad, version 5.2.008.0222), and data are expressed as relative mRNA quantity.

### 5.8. Total GSH Content Determination and Enzymatic Activity Assays

The determination of total GSH content and enzymatic activities was performed on hepatic cytosolic fractions obtained by differential ultracentrifugation [59]. Bovine serum albumin was used as reference standard to quantify protein content [60]. GSH was measured on TCA deproteinized samples using the Elmann’s reagent as previously reported [61], and the results are expressed as μmol of GSH per g of liver. Total GST, GST μ-class, and GST α-class activities were tested by monitoring the continuous increase at λ = 340 nm of the GSH conjugates of 1 mM 1-chloro, 2,4-dinitrobenzene (CDNB), 1 mM 3,4-dichloronitrobenzene (DCNB) [62,63], and tert-butyl hydroperoxide 1.5 mM [64], respectively. The activity of DT-diaphorase was measured following the continuous reduction of 2,6-dichloroindophenol at 600 nm [65]. All assays were performed under Vmax conditions and were linear in terms of protein concentration and time. The results are expressed as nmol/min per mg of protein.

### 5.9. Statistical Analysis

Data are expressed as mean ± standard error of the mean (SEM). Statistical analysis was run on the GraphPad Prism 7.03 software (GraphPad Software, San Diego, CA, USA). The D’Agostino and Pearson normality omnibus test was applied to verify the normal distribution of the data (n = 8 for each experimental group). The one-way or the two-way analysis of variance (ANOVA), followed by the Tukey’s post hoc test, was used to check the significant differences among groups. The one-way ANOVA was applied to the concentrations in the excreta and the calculated absorption rate of AFBs. The other parameters were analyzed through the two-way ANOVA, considering the exposure to the mycotoxin (AFB1) and the presence/absence of the MyDA as the factors. A two-sided *p*-value of less than 0.05 was considered statistically significant.

## Figures and Tables

**Table 1:** Growth performance and productive parameters of broiler chickens fed the experimental diets (n = 4; mean ± SEM).

Parameters	Treatments						*p*-Value
	BD	SeOX	CHS	AFB1	AFB1 + SeOX	AFB1 + CHS	
Initial body weight (d23) (g)	1011 ± 22	1004 ± 15	999 ± 25	987 ± 29	996 ± 22	997 ± 34	0.802
Final body weight (d32) (g)	1944 ± 41	1965 ± 33	1954 ± 42	1944 ± 66	1976 ± 28	1949 ± 43	0.841
ADG 23–32 days of age (g)	118 ± 4.1	121 ± 2.9	120 ± 3.8	120 ± 3.6	123 ± 1.6	120 ± 5.2	0.574
ADFI 23–32 days of age (g)	144 ± 5.8	144 ± 2.5	145 ± 7.5	144 ± 7.1	149 ± 8.1	144 ± 11	0.776
FCR 23–32 days of age	1.53 ± 0.02	1.50 ± 0.03	1.51 ± 0.05	1.52 ± 0.02	1.52 ± 0.07	1.51 ± 0.08	0.936

ADG, average daily gain; ADFI, average daily feed intake; FCR, feed conversion ratio; BD, basal diet; AFB1, aflatoxin B1 group; SeOX, SeOX MyDA group; CHS, CHS MyDA group; AFB1 + SeOX, aflatoxin B1 plus SeOX group; AFB1 + CHS, aflatoxin B1 plus CHS group.

**Table 2:** GSH content and enzymatic activities of total GST, GST α-class, GST μ-class, and DT-diaphorase in liver samples from experimental broilers.

Parameter	Treatments					
	BD	SeOX	CHS	AFB1	AFB1 + SeOX	AFB1 + CHS
GSH (μmol/g liver)	2.23 ± 0.24	2.29 ± 0.16	2.38 ± 0.27	2.15 ± 0.17	2.51 ± 0.13	1.77 ± 0.11
Total GST (nmol/min/mg protein)	504 ± 26.84	595.8 ± 25.7	586 ± 38.82	563 ± 26.28	551 ± 33.48	545.2 ± 25.26
α-class GST (nmol/min/mg protein)	45.1 ± 2.86	40.75 ± 1.04	42.57 ± 1.37	40.3 ± 2.86	43.29 ± 1.8	39.52 ± 1.77
µ-class GST (nmol/min/mg protein)	1.04 ± 0.06	1.43 ± 0.11	1.24 ± 0.15	1.50 ± 0.22	1.19 ± 0.18	1.14 ± 0.17
DT-diaphorase (nmol/min/mg protein)	367.8 ± 14.69	390.4 ± 6.42	404 ± 13.56	371.4 ± 18.71	396.5 ± 23.01	376.2 ± 16.88

BD, basal diet; AFB1, aflatoxin B1 group; SeOX, SeOX MyDA group; CHS, CHS MyDA group; AFB1 + SeOX, aflatoxin B1 plus SeOX group; AFB1 + CHS, aflatoxin B1 plus CHS group. Data are expressed as mean ± SEM, n = 8.

## Data Availability

The original contributions presented in the study are included in the article/Supplementary Material, and further inquiries can be directed to the corresponding author/s.

## Supplementary Materials

The following supporting information can be downloaded at www.mdpi.com/article/10.3390/toxins17020082/s1. Table S1: Primers for quantitative real-time PCR (qRT-PCR); Table S2: Matrix-matched regression curves and related R2 and SSE (%) values; Table S3: Limit of detection (LOD, S/N = 3) and limit of quantification (LOQ, S/N = 10) calculated for AFB1, AFB2, AFG1, AFG2, AFM1, AFM2, AFQ1, and AFL in excreta samples; Table S4: Recoveries of AFB1,AFB2, AFG1, AFG2, AFM1, AFM2, AFQ1, and AFL in excreta samples spiked at two different concentration levels (5 and 25 ng/g).
