# Supplementary material for: Innovative Mycotoxin Detoxifying Agents Decrease the Absorption Rate of Aflatoxin B1 and Counteract the Oxidative Stress in Broiler Chickens Exposed to Low Dietary Levels of the Mycotoxin
Source: Toxins (Basel). 2025 Feb 10;17(2):82. doi: 10.3390/toxins17020082 (PMC11861810; doi:10.3390/toxins17020082)
Supplement: Supplementary file 1 [file toxins-17-00082-s001.zip › toxins-3274099-supplementary.pdf]

Supplementary Materials: Innovative Mycotoxin Detoxifying Agents Decrease the Absorption Rate of Aflatoxin B1 and Counteract the Oxidative Stress in Broiler Chickens Exposed to Low Dietary Levels of the Mycotoxin

Matteo Cuccato, Neenu Amminikutty, Veronica Spalenza, Vanessa Conte, Stefano Bagatella, Donato Greco, Vito D'Ascanio, Francesco Gai, Achille Schiavone, Giuseppina Avantaggiato, Carlo Nebbia, and Flavia Girolami

Table S1. Primers for Quantitative Real-Time PCR (qRT-PCR).

Gene	Accession no.	Sequence	Melting temperature (°C)	Amplicon size (bp)
CYP1A1	NM_205147.1	FW: GATGTCCGCGTCCAACCC	61.32	86
		REV: GCGGTTGTCACGGTGTCAA	60.47	
CYP1A2	NM_205146.2	FW: CGCAGATCCCAAACGAGAAG	60.61	76
		REV: GCGGTTGTCACGGTGTCAA	60.47	
CYP2A6	KX687985	FW: CCCTCTCCTAAACAGATGCG	57.39	149
		REV: TTGCTGTCTCCCATCCTGC	58.81	
CYP2H1	NM_001001616.1	FW: TCCTTCCCCTTAATGTTCCACA	61.08	98
		REV: GGGAGACAGCAAAGGGAATATC	59.61	
CYP3A4	NM_001329508.2	FW: TGGTAGTCATGATCCCAGCC	57.77	113
		REV: GGGGTCAATGTTCTCTCCGT	58.53	
EPHX1	XM_419386.6	FW: TCCTCAATGCGTTTCTACAAAGA	59.62	107
		REV: TCATTAGGAAAGGAGGCAATGC	61.14	
EPHX2	NM_001033645.1	FW: CAAGGGCATGGAGGAGTGG	60.74	80
		REV: GCCTCTCCATTTGTGTCCAA	58.05	
GSTA1	NM_001001777.1	FW: TTTTAGCGGTGGAAGAGTCG	58.15	86
		REV: GGGGATATTGCTTGTTCTTGCT	60.3	
GSTA2	NM_001001776.1	FW: AGCAGCCGATGTGAAAGAAAA	60.15	115
		REV: GCCAACAAGATAATCCTGACCA	59.28	
GSTA4	XM_015284816.2	FW: AGAGAGCCCTGATCGACATG	57.16	130
		REV: CTCTCTGTTGCCTTCTCTGC	54.79	
GSTM2	NM_205090.1	FW: CAACCTGAGCCAATTCCTGC	60.41	104
		REV: GCGCCGTGTACCAGAAAAT	58.5	
Nrf2	NM_205117	FW: AATCAAACTCAGCCACCCAG	57.35	142
		REV: CAGCCAGGTTGTCGTTTTCA	59.3	
CAT	NM_001031215.2	FW: GGCACTGCTGGACAAATACA	56.66	71
		REV: AAGTGGCTTGCGTGTATGTC	55.94	
GPX1	NM_001277853.2	FW: TTCGGGCACCAGGAGAACGC	67.68	91
		REV: TGGTGAAGTTGGGTTTGAAGC	60.04	
SOD1	NM_205064.1	FW: GGGAGGAGTGGCAGAAGTAG	56.2	115
		REV: CCCTCTACCCAGGTCATCAC	56.35	
SOD2	NM_204211.1	FW: GGAGCAGGGACGTCTACAAA	57.54	81
		REV: CCCAGCAATGGAATGAGACC	59.95	
ABCB1	NM_204894.1	FW: ACAACAGTCGGGAGGTGTC	54.62	123
		REV: GCTGTGTTCCCTTGTCTCCT	56.32	
ABCC2	XM_015288821.2	FW: TCCTTGTTCTTTGTCACCACA	59.22	122
		REV: AGTAGGCAGACACGCGATAA	58.39	
ABCG2	NM_001328490.1	FW: TCCTTGTTCTTTGTCACCACA	56.08	124
		REV: AGTAGGCAGACACGCGATAA	56.02	
PGK2	NM_204985.2	FW: CTGCTGGCTTCCTGATGAA	56.62	103
		REV: TCCTGAACTTTAGCTCCTCCA	56.82	
RPS7	XM_001234708.4	FW: GCCCAAGCCAACGAGAAAA	61.0	138
		REV: TTTACGCGGATTCTCTTGCC	60.48	

Italicized gene names indicate the internal control genes used as references.

Table S2. Matrix-matched regression curves and related R2 and SSE (%) values.

Toxins	Calibration range	n° exp. points	Matrix-matched calibration curve	R^2	SSE (%)
AFB1	0.1-25 ng/mL	5	y=0.84x+0.04	0.998	96
AFB2			y=2.97x+0.10	0.999	94
AFG1			y=0.40x+0.004	0.998	97
AFG2			y=0.54x-0.05	0.999	92
AFM1			y=0.35x+0.02	0.999	93
AFM2			y=1.11x-0.008	0.998	96
AFQ1			y=0.16x+0.01	0.999	102
AFL			y=0.25x+0.02	0.999	96

SSE(%) = signal suppression/enhancement (matrix calibration line slope/standard calibration line slope *100).

Table S3. Limit of detection (LOD, S/N=3) and limit of quantification (LOQ, S/N = 10) calculated for AFB1, AFB2, AFG1, AFG2, AFM1, AFM2, AFQ1 and AFL in excreta samples.

	ng AF/g feces (ppb)							
	AFB1	AFB2	AFG1	AFG2	AFM1	AFM2	AFQ1	AFL
**LOD**	1	1	1	0.3	0.2	2	0.5	2
**LOQ**	3	3	3	1	1	7	2	7

Table S4. Recoveries of AFB1, AFB2, AFG1, AFG2, AFM1, AFM2, AFQ1 and AFL in excreta samples spiked at 2 different concentration levels (5 and 25 ng/g).

Spiking level (ng/g)	Recovery, % (RSD,%, *n*=3)							
	AFB1	AFB2	AFG1	AFG2	AFM1	AFM2	AFQ1	AFL
**5**	55 (3)	100 (4)	58 (4)	104 (1)	101 (6)	104 (5)	104 (1)	104 (2)
**25**	41 (3)	108 (4)	40 (1)	109 (1)	112 (1)	101 (2)	110 (1)	105 (2)

Analysis of AF was performed using the optimized analytical method and quantification was carried out using the standard calibration line. RSD = relative standard deviation.
